# Predicting transcription factor binding sites using local over-representation and comparative genomics

**DOI:** 10.1186/1471-2105-7-396

**Published:** 2006-08-31

**Authors:** Matthieu Defrance, Hélène Touzet

**Affiliations:** 1 LIFL, UMR CNRS 8022, Université des Sciences et Technologies de Lille, Villeneuve d'Ascq, France

## Abstract

**Background:**

Identifying *cis*-regulatory elements is crucial to understanding gene expression, which highlights the importance of the computational detection of overrepresented transcription factor binding sites (TFBSs) in coexpressed or coregulated genes. However, this is a challenging problem, especially when considering higher eukaryotic organisms.

**Results:**

We have developed a method, named TFM-Explorer, that searches for locally overrepresented TFBSs in a set of coregulated genes, which are modeled by profiles provided by a database of position weight matrices. The novelty of the method is that it takes advantage of spatial conservation in the sequence and supports multiple species. The efficiency of the underlying algorithm and its robustness to noise allow weak regulatory signals to be detected in large heterogeneous data sets.

**Conclusion:**

TFM-Explorer provides an efficient way to predict TFBS overrepresentation in related sequences. Promising results were obtained in a variety of examples in human, mouse, and rat genomes. The software is publicly available at .

## Background

The computational identification of functional transcription factor (TF) binding sites (TFBSs) from a nucleotide sequence alone is difficult. TFBSs are usually short (around 5–15 bases) and degenerate, and hence potential binding sites can occur very frequently by chance, leading to a high level of false positive in the predicted sites. Wasserman and Sandelin have termed this the *futility theorem*, since nearly 100% of predicted TFBSs have no function *in vivo *[1]. Solving this problem is crucial for mammalian genomes that contain large noncoding regions.

Phylogenetic footprinting can significantly increase the accuracy of TFBSs predictions. If a region is conserved between sequences from distantly related organisms, it is likely to be subject to greater selection pressure and to have a biological role. Phylogenetic footprinting methods are based on the assumption that TFBSs are located in conserved regions that can be detected by alignment algorithms. A current limitation for mammalian organisms is that when nothing is known about the motif, the number of orthologous sequences at the correct evolutionary distance needs to be high [2].

Another potentially fruitful approach for improving the accuracy of TFBS prediction is to use a set of coexpressed genes. The rationale behind this approach is that coexpressed genes should contain common *cis*-regulatory elements in their noncoding sequences, with the number of motifs for these elements being greater than what would be expected by chance. The application of Gibbs sampling algorithms [3] and combinatorial algorithms [4,5] to the problem of *de novo *motif inference has proven successful in identifying *cis*-regulatory elements in bacterial and yeast genomes, but *de novo *motif discovery in higher eukaryotic genomes remains an unsolved challenge [6]. It is also possible to focus on overrepresented motifs modeled by known profiles, such as position weight matrices (PWMs) [7,8]. Large databases of PWMs are available, including JASPAR [9] and TRANSFAC [10]. Several tools for evaluating the significance of a set of putative TFBSs modeled by PWMs have recently been made available (e.g., MSCAN, OTFBS, TOUCAN [11-14]). These programs can handle sequences coming from either one or multiple species, although in the latter case the same background model is used for all sequences. oPOSSUM [15] makes an exception: it combines a precomputed database of conserved TFBSs in human and mouse promoters, and uses statistical methods to identify overrepresented sites in a set of coexpressed genes.

In this paper, we present a new method that searches for locally overrepresented TFBSs in a set of coregulated genes, which we have named TFM-Explorer ("TF matrix explorer"). TFM-Explorer associates motif overrepresentation with comparative genomics, allowing for multiple species to be included. One novel feature of the method is that it takes advantage of the spatial conservation of *cis*-regulatory elements, when it exists. Often, the distance from *cis*-regulatory elements to the transcription start site (TSS) plays an essential role in the control of the gene. The activity of basal or general TFs, such as GC-box binding or TATA-box binding proteins, is strongly conditioned by the distance from the binding site to the TSS and, as far as we know, no existing tools exploit this information.

More precisely, TFM-Explorer relies on three main principles. The first is that the background distribution used to assess the statistical significance of overrepresented motifs is a local model that depends on the location on the sequence with respect to the TSS. This allows us to cope with large heterogeneous regulatory regions, including proximal *cis*-regulatory elements as well as distal enhancers. Second, it is possible to combine background models between sequences, which makes the method able to cope with multiple species. In contrast with other phylogenetic footprinting approaches, genes do not need to be orthologous, and conserved TFBSs are not expected to be surrounded by similar regions that can be easily aligned. Lastly, we use spatial conservation as supplementary information, for which we have developed an algorithm that is able to identify the portion of sequences with local overrepresentation without prior knowledge of either the size or the location of the involved region. This allows us to infer short regions exhibiting a local signal, as well as large regions when we have to identify *cis*-regulatory motifs that show no spatial conservation.

## Implementation

The input to TFM-Explorer is a set of upstream sequences that are expected to share *cis*-regulatory elements. These sequences may come from several species, and need not be aligned beforehand. The only requirement is that the location of the TSS needs to be known for each sequence.

TFM-Explorer then uses PWMs available in the TRANSFAC or JASPAR database to search for locally overrepresented TFBSs, and outputs a list of regions that show significant TFBS overrepresentation. The search algorithm includes three steps that are discussed in the following subsections: (1) localizing all potential TFBSs for a database of PWMs, (2) identifying windows showing an overrepresentation for a given PWM, and (3) assessing the statistical significance of the regions found at the previous stage.

### Localizing all potential TFBSs

The method initially identifies all potential TFBSs for a set of PWMs given by a database of profiles. TFBSs are usually selected using a score cutoff that expresses the probability in a target model – the profile – compared to the probability of the motif appearing in a background model [8,16]. Therefore, the selection of putative positions is highly dependent on the choice of background model. This point is crucial for higher eukaryotic organisms due to the variability of the sequence content in upstream regions [17], which makes it difficult to build uniform models for the entirety of upstream regions. We follow the approach recently proposed by Huang et al., which is using a threshold based on the parameters determined by the genomic context [18]. Given a PWM, we obtain for each sequence a set of putative TFBSs in which overlapping occurrences are removed; these are referred to as *hits*.

### Extracting regions with a high density of TFBSs

The second step involves discovering regions showing *local *overrepresentation of hits for a given PWM. All existing methods implicitly rely on *global *overrepresentation, looking for motifs that have a significant number of occurrences among the entire set of sequences. But a short signal, covering a few dozen bases, may be overwhelmed by a flat distribution of hits in the neighborhood. In this case, the result depends on the size of the input sequences: the signal is found if the sequences are short, but is lost if they are long. This is why we introduce a strategy that is not influenced by the length of the data set, and that is able to recover short but significant regions in large sequences.

One solution is to employ a sliding window technique, applying statistical analysis to each window along the set of sequences. The main drawback of this approach is that the result can be highly dependent on the window size, and testing several window sizes is time-consuming. To circumvent this problem, we developed a heuristic algorithm based on a positional scoring scheme that takes into account the expected frequency of each hit according to both its position in the upstream sequence and the corresponding species. Let *N *be the total number of sequences, *i *be a position relative to the TSS, and *k*_*i *_be the number of sequences having a hit at position *i*. The associated score *s*_*i *_is defined as follows:

si=(∑n=1Nλin∑n=1Nμin)kieN(∑n=1Nμin−∑n=1Nλin)
 MathType@MTEF@5@5@+=feaafiart1ev1aaatCvAUfKttLearuWrP9MDH5MBPbIqV92AaeXatLxBI9gBaebbnrfifHhDYfgasaacH8akY=wiFfYdH8Gipec8Eeeu0xXdbba9frFj0=OqFfea0dXdd9vqai=hGuQ8kuc9pgc9s8qqaq=dirpe0xb9q8qiLsFr0=vr0=vr0dc8meaabaqaciaacaGaaeqabaqabeGadaaakeaacqWGZbWCdaWgaaWcbaGaemyAaKgabeaakiabg2da9maabmGabaWaaSaaaeaadaaeWaqaaGGaciab=T7aSnaaDaaaleaacqWGPbqAaeaacqWGUbGBaaaabaGaemOBa4Maeyypa0JaeGymaedabaGaemOta4eaniabggHiLdaakeaadaaeWaqaaiab=X7aTnaaDaaaleaacqWGPbqAaeaacqWGUbGBaaaabaGaemOBa4Maeyypa0JaeGymaedabaGaemOta4eaniabggHiLdaaaaGccaGLOaGaayzkaaWaaWbaaSqabeaacqWGRbWAdaWgaaadbaGaemyAaKgabeaaaaGccqWGLbqzdaahaaWcbeqaaiabd6eaonaabmGabaWaaabmaeaacqWF8oqBdaqhaaadbaGaemyAaKgabaGaemOBa4gaaaqaaiabd6gaUjabg2da9iabigdaXaqaaiabd6eaobGdcqGHris5aSGaeyOeI0YaaabmaeaacqWF7oaBdaqhaaadbaGaemyAaKgabaGaemOBa4gaaaqaaiabd6gaUjabg2da9iabigdaXaqaaiabd6eaobGdcqGHris5aaWccaGLOaGaayzkaaaaaaaa@6712@

where λin
 MathType@MTEF@5@5@+=feaafiart1ev1aaatCvAUfKttLearuWrP9MDH5MBPbIqV92AaeXatLxBI9gBaebbnrfifHhDYfgasaacH8akY=wiFfYdH8Gipec8Eeeu0xXdbba9frFj0=OqFfea0dXdd9vqai=hGuQ8kuc9pgc9s8qqaq=dirpe0xb9q8qiLsFr0=vr0=vr0dc8meaabaqaciaacaGaaeqabaqabeGadaaakeaaiiGacqWF7oaBdaqhaaWcbaGaemyAaKgabaGaemOBa4gaaaaa@3154@ and μin
 MathType@MTEF@5@5@+=feaafiart1ev1aaatCvAUfKttLearuWrP9MDH5MBPbIqV92AaeXatLxBI9gBaebbnrfifHhDYfgasaacH8akY=wiFfYdH8Gipec8Eeeu0xXdbba9frFj0=OqFfea0dXdd9vqai=hGuQ8kuc9pgc9s8qqaq=dirpe0xb9q8qiLsFr0=vr0=vr0dc8meaabaqaciaacaGaaeqabaqabeGadaaakeaaiiGacqWF8oqBdaqhaaWcbaGaemyAaKgabaGaemOBa4gaaaaa@3156@ are the parameters of the Poisson distribution for sequence *n *at position *i *in the target and background models, respectively, and *s*_*i *_expresses ratio between the probabilities of observing *k*_*i *_hits in the target and background models when the distribution of hits is approximated by a Poisson distribution. In practice, μin
 MathType@MTEF@5@5@+=feaafiart1ev1aaatCvAUfKttLearuWrP9MDH5MBPbIqV92AaeXatLxBI9gBaebbnrfifHhDYfgasaacH8akY=wiFfYdH8Gipec8Eeeu0xXdbba9frFj0=OqFfea0dXdd9vqai=hGuQ8kuc9pgc9s8qqaq=dirpe0xb9q8qiLsFr0=vr0=vr0dc8meaabaqaciaacaGaaeqabaqabeGadaaakeaaiiGacqWF8oqBdaqhaaWcbaGaemyAaKgabaGaemOBa4gaaaaa@3156@ is determined from a large sets of sequences, and λin
 MathType@MTEF@5@5@+=feaafiart1ev1aaatCvAUfKttLearuWrP9MDH5MBPbIqV92AaeXatLxBI9gBaebbnrfifHhDYfgasaacH8akY=wiFfYdH8Gipec8Eeeu0xXdbba9frFj0=OqFfea0dXdd9vqai=hGuQ8kuc9pgc9s8qqaq=dirpe0xb9q8qiLsFr0=vr0=vr0dc8meaabaqaciaacaGaaeqabaqabeGadaaakeaaiiGacqWF7oaBdaqhaaWcbaGaemyAaKgabaGaemOBa4gaaaaa@3154@ is obtained by scaling μin
 MathType@MTEF@5@5@+=feaafiart1ev1aaatCvAUfKttLearuWrP9MDH5MBPbIqV92AaeXatLxBI9gBaebbnrfifHhDYfgasaacH8akY=wiFfYdH8Gipec8Eeeu0xXdbba9frFj0=OqFfea0dXdd9vqai=hGuQ8kuc9pgc9s8qqaq=dirpe0xb9q8qiLsFr0=vr0=vr0dc8meaabaqaciaacaGaaeqabaqabeGadaaakeaaiiGacqWF8oqBdaqhaaWcbaGaemyAaKgabaGaemOBa4gaaaaa@3156@. This positional score is then incorporated into an incremental score:

*S*_*i*_*= *max(0,*S*_*i*-1 _+ ln *s*_*i*_)

**Figure 1:**
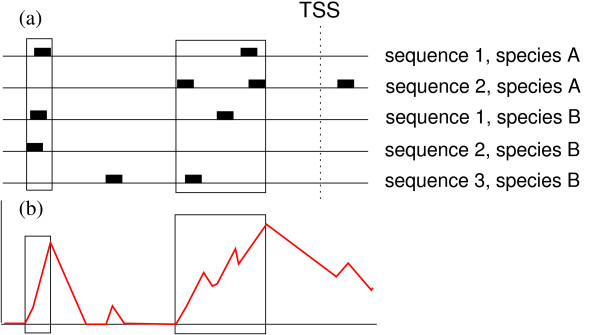
**Score profile and window extraction**. Example of the score used to predict windows with a significant overrepresentation of TFBSs. Panel (*a*) shows the predicted TFBSs (black boxes) along the upstream sequences of five genes that come from two species. Panel (*b*) shows the evolution of the cumulative score computed for a given PWM with those sequences. Local overrepresentations detected by the algorithm are represented by boxes.

High-scoring regions are retained as candidate regions for the next step of the method (see Figure 1). This scoring scheme leads to a very efficient search algorithm. Sequences are scanned "on the fly", which enables large-scale data analysis. One point worth noting is that this scoring strategy allows windows to be extracted without knowing *a priori *whether they are proximal or distal, short or long. Moreover, as shown in the Results section, the method can also detect several consecutive windows corresponding to collaborative TFs.

### Evaluating the statistical significance of overrepresentation

The final step of the method consists of evaluating the statistical significance of the candidate windows that have been identified at the previous stage. We have to determine whether the number of hits for each window could be observed by chance. To this end, we compute a probability called the P-value: given a PWM *M *and a window [*i, j*] containing *k *hits for *M*, the P-value is defined as the probability of observing at least *k *hits in window [*i, j*] with the same combination of background models (i.e., the same number of sequences coming from each species).

For each sequence, the distribution of hits in window [*i, j*] is approximated by a Poisson distribution, whose parameter is derived from the region [*i, j*] in the background model. We used a goodness-of-fit test to evaluate the validity range of this approximation of the hit-count distribution. For all JASPAR and TRANSFAC vertebrate matrices, we computed the chi-square goodness-of-fit for different locations and sizes of window applied to a large set of human gene upstream sequences. Table 1 indicates that the majority of PWMs passed the test.

To determine the significance of the window for the entire set of sequences, we sum the distributions to handle the combination of models for sequences coming from several species.

Assuming that motif occurrences are mutually independent, the P-value can be defined as follows:

P(X≥k)=1−∑z=0k−1(|w|∑n=1Nμn)zz!e−|w|∑n=1Nμn
 MathType@MTEF@5@5@+=feaafiart1ev1aaatCvAUfKttLearuWrP9MDH5MBPbIqV92AaeXatLxBI9gBaebbnrfifHhDYfgasaacH8akY=wiFfYdH8Gipec8Eeeu0xXdbba9frFj0=OqFfea0dXdd9vqai=hGuQ8kuc9pgc9s8qqaq=dirpe0xb9q8qiLsFr0=vr0=vr0dc8meaabaqaciaacaGaaeqabaqabeGadaaakeaacqWGqbaucqGGOaakcqWGybawcqGHLjYScqWGRbWAcqGGPaqkcqGH9aqpcqaIXaqmcqGHsisldaaeWbqaamaalaaabaGaeiikaGIaeiiFaWNaem4DaCNaeiiFaW3aaabmaeaaiiGacqWF8oqBdaWgaaWcbaGaemOBa4gabeaaaeaacqWGUbGBcqGH9aqpcqaIXaqmaeaacqWGobGta0GaeyyeIuoakiabcMcaPmaaCaaaleqabaGaemOEaOhaaaGcbaGaemOEaONaeiyiaecaaiabdwgaLnaaCaaaleqabaGaeyOeI0IaeiiFaWNaem4DaCNaeiiFaW3aaabmaeaacqWF8oqBdaWgaaadbaGaemOBa4gabeaaaeaacqWGUbGBcqGH9aqpcqaIXaqmaeaacqWGobGta4GaeyyeIuoaaaaaleaacqWG6bGEcqGH9aqpcqaIWaamaeaacqWGRbWAcqGHsislcqaIXaqma0GaeyyeIuoaaaa@6459@

where *μ*_*n *_is the parameter of the Poisson distribution for the *n*^th ^sequence in a window [*i, j*], and |*w*| is the window width.

## Results

To evaluate the efficiency of TFM-Explorer, we performed three case studies involving human, mouse, and rat data sets: Rel/NF-*κ*B target genes, muscle-specific genes, and genes coding for histones. In the first part of this section we compare the results of TFM-Explorer with those obtained by using three existing tools that are also based on PWM overrepresentation: OTFBS [13], TOUCAN [14], and oPOSSUM [15]. In the last part, we describe the results of applying TFM-Explorer to random data sets to evaluate its robustness to noise.

**TOUCAN **is an integrated suite of software for discovering *cis*-regulatory elements in a set of related genes. For our purposes, we first used MotifScanner [14] to predict potential TFBSs and then performed the statistical overrepresentation analysis. MotifScanner searches for potential TFBSs in a set of sequences by maximizing the probability of observing those sequences in a mixed model composed of motif instances and a Markovian background model. This allowed us to predict instances of potential TFBSs for all the TRANSFAC vertebrate PWMs in our input sequences. The statistical overrepresentation tool was then used to extract PWMs that had significantly overrepresented instances. This statistical overrepresentation is based upon a binomial P-value that is the probability of finding at least the observed number of TFBSs instances in a precomputed background model. We used version 2.2.5 of TOUCAN with the TRANSFAC Public 7.0 Vertebrate PWMs database and the EPD(3) Markovian model to run MotifScanner, leaving other parameters unchanged. The prior-frequency file epd_vertebrates_499_prior0.1.freq was then used to compute the statistical overrepresentation.

**OTFBS **searches for overrepresented motifs of known TFs from a set of related sequences. Like TOUCAN it proceeds in two steps to extract the most significant TFs from among all TRANSFAC PWMs: it first searches for potential TFBSs using the Matinspector algorithm [19] and then computes a P-value for each PWM using a binomial significance test. Version 1.0 of OTFBS was used simply by pasting our sequences (since no options were available).

**oPOSSUM **uses different methods to identify overrepresented TFBSs. It combines a precomputed database of conserved TFBSs in human and mouse promoters with statistical methods for detecting overrepresentation (one-tailed Fisher exact probability analysis and Z-score) to identify overrepresented TFBSs in the input gene promoters. Version 1.3 of oPOSSUM was applied to all the data sets using JASPAR vertebrate PWMs (no other PWM database was available) and default parameters. Results were sorted based on the Fisher P-value.

We used TFM-Explorer with the default parameters. All upstream sequences were retrieved from the UCSC Genome Browser [20] (assemblies hg18, mm8, and rn3) using RefSeq identifiers.

### Skeletal-muscle-specific genes

The first example involves a set of genes with skeletal-muscle-specific expression. This is an up-to-date version of the reference compilation of Wasserman and Fickett [21], which has been widely used to assess the accuracy of TFBS prediction programs [see Additional file 1]. The data set contained nine human genes. Early steps in skeletal muscle development are controlled by combinatorial interactions between members of the MyoD family of basic helix-loop-helix TFs (MyF) and TFs from the MADS family, with the myocyte enhancer factor-2 (MEF2) and the serum response factor (SRF) [22]. Other TFs, such as TEF, MZF, and Sp1, are also known to contribute to skeletal-muscle-specific expression.

Table 2 reports the predictions of TFM-Explorer, Toucan, OTFBS, and oPOSSUM for this data set. The three most significant TFs ranked by TFM-Explorer are SRF, MEF-2, and MZF_1, all of which correspond to factors that are known to be involved in the regulation of muscle-specific genes. Under the same conditions, OTFBS predicted two TFs (MZF1 and MEF2) involved in the regulation of muscle-specific genes, but only at the second and third rank. oPOSSUM and TOUCAN predicted only one factor (MEF2) that is known to contribute to muscle-specific expression among the top five factors. Note that oPOSSUM achieved this result by using supplementary orthologous mouse genes and by taking advantage of conservation between human and mouse promoter sequences.

### Rel/NF-*κ*B target genes

The second data set comprised Rel/NF-*κ*B human target genes. Rel/NF-*κ*B TFs are involved in inflammatory and immunizing mechanisms, as well as apoptosis. Five regulatory proteins of this family are known in vertebrate organisms: c-Rel, RelA (p65), RelB, NF-*κ*B1 (p50), and NF-*κ*B2 (p52); and they share similar binding sites. This corresponds to six PWMs in the TRANSFAC database: CREL_01, NFKAPPA50_01, NFKAPPAB65_01, NFKB_Q6, NFKAPPAB_01, and NFKB_C. All of these PWMs contain the consensus pattern 5'-GGGRNYYYCC-3'.

A set of 99 human target genes with experimentally verified binding sites was compiled from the literature [23] [see Additional file 2]. In order to test how the sequence length influenced the predictions for each program, we selected both large and short regulatory sequences for these genes: those 2 kb upstream, and those 5 kb upstream and 5 kb downstream of the TSS were used. We first applied TFM-Explorer to those sequences, searching exhaustively for all vertebrate PWMs in TRANSFAC (243 matrices). Nearly identical results were obtained by TFM-Explorer for short and long sequence sets. This shows one advantage of the local approach of TFM-Explorer: its ability to identify several local regions in a large data set without compromising the sensitivity.

The top-ten windows identified by TFM-Explorer with the associated TFs are listed in Table 3. The first notable observation is that all windows are located around the TSS, which is a region rich in *cis*-regulatory elements. Second, the six PWMs corresponding to TFs from the Rel/NF-*κ*B family are all present in the list, and the location of the associated window in the promoter is consistent with the location of the experimentally verified binding sites [23].

The remaining TFs do not correspond to experimentally verified binding sites for this data set. However, except for CDXA_01, there are many indications that they are biologically valid. Besides the Rel/NF-*κ*B factors, TFM-Explorer identified a short window for TATA-box binding proteins located 40 bp upstream of the TSS. Both the size and position of the window are characteristic of this factor. The prediction indicates that 40% of genes in the data set contain a TATA-box, compared to 32% in the human genome [24]. Fast inducible genes (such as genes mediated by Rel/NF-*κ*B) frequently contain a strong TATA-box in their core promoter. In contrast, TATA-box-less genes tend to be expressed at a low and constant rate.

Therefore, this relative abundance of TATA-boxes in the core promoter is an expected property for this data set. Another TF family detected by TFM-Explorer is Spl, which is a zinc-finger TF that binds to GC-boxes. Once again, the position of the window ([-94 : -43]) is consistent with the information on this factor published in the literature. Several Rel/NF-*κ*B target genes that are present in the data set show promoter organization containing functional GC-boxes, such as MnSod [25] and interleukins [26].

In order to compare our results with predictions made by other tools, we applied OTFBS, TOUCAN, and oPOSSUM to the data set with both short and long sequences (Table 3). One of the most noticeable results is that while all the programs performed relatively well on relatively short sequences (2 kb), this was not the case with longer sequences (5 kb upstream and 5 kb downstream). In this last case, only oPOSSUM was able to give reliable predictions. Moreover, oPOSSUM produced similar results for the long and short sequences, since it searches for TFBSs only in regions that are conserved across human and mouse.

### Histone genes

Histone proteins are at the heart of the chromatin structure in the eukaryotic cell nucleus. They act as a spool and help in packing DNA by wrapping it around. They also play an important role in transcriptional regulation. They are divided into five classes: H1, H2A, H2B, H3, and H4. These proteins (particularly H3 and H4) are known to be highly conserved evolutionarily. Four of the functional motifs that are known to be involved in H3 regulation have been clearly identified: CCAAT-box, Oct-1 box, GC-box, and AC-Box.

Excluding the AC-box (for which no entry was found in TRANSFAC), the corresponding matrices in TRANSFAC database are as follows: NFY_01, NFY_C, NFY_Q6, CAAT_01, and CAAT_C for CCAAT-boxes; OCT1_Q6, OCT1_C, and OCT1_0* for Oct-1 boxes; and SP1_01 and SP1_Q6 for GC-boxes.

One advantage of TFM-Explorer is its ability to cope with heterogeneous sets of genes. We evaluated the impact of using genes from a related species, with a set of 19 H3 genes compiled from [27] [see Additional file 3], comprising 11 human, 7 mouse, and 1 rat genes. Sequences of 2 kb that were upstream of the TSS were submitted against all TRANSFAC vertebrate matrices to TFM-Explorer. Table 4 indicates that two functional motifs (CCAAT-box and Oct-1 box) known to be involved in the regulation of H3 genes were predicted, which correspond to the TRANSFAC matrices NFY_C and NFY_Q6, and OCT1_04 and OCT1_07, respectively.

Among the top-five predictions of TFM-Explorer, only one matrix, XFD1_01, was unlikely to be found. An explanation for this false positive prediction comes from the profile of XFD1_02. It appears that it is likely to find occurrences of XFD1_01 where OCT1_04 or OCT1_07 match, because of the similarity between their profiles. In TFM-Explorer, we added the ability to compare two different predictions and to identify such redundant or biased results. The comparison was performed on the basis of the proportion of overlapping hits. We also computed the theoretical rate of overlapping hits using a previously reported similarity measure [28]. In this case, a large number of XFD1_01 TFBSs actually overlap with OCT1_07 and OCT1_04 TFBSs (37% and 53%, respectively). A similar conclusion can be drawn for PBX1_02 and matrices corresponding to the CCAAT-box. The predictions made by TOUCAN and OTFBS are listed in Table 4; oPOSSUM is not included since it was unable to produce results from this data set.

### Robustness to noise

**Figure 2:**
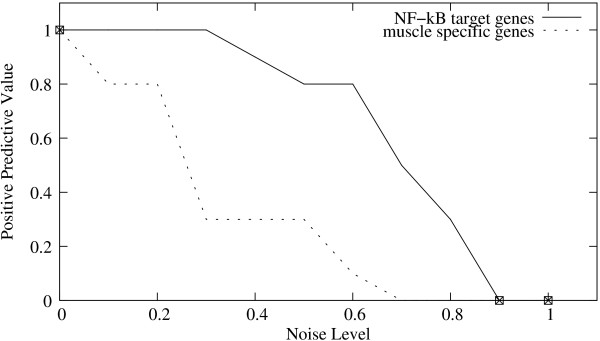
**Influence of noise on the positive predictive value**. Starting from the Rel/NF-*κ*B and muscle data sets, an increasing number of actual sequences were replaced by random sequences. The noise level represents the proportion of sequences for the given set that have been randomly selected in the genome. The positive predictive value corresponds to the proportion of valid predictions (the most significant extracted TF is known to be involved in the regulation of the reference set).

In order to test the robustness of TFM-Explorer, we also measured its ability to detect regulation signals in a noisy environment. We constructed artificial data sets with various noise levels in the following way: starting with homogeneous data sets (the NF-*κ*B target genes and muscle-specific data sets presented above), we replaced from 10% to 90% of the reference sequences with randomly selected genes, generating 100 such data sets for each noise level. The percentage of correct predictions is reported in Figure 2. The prediction was considered to be correct when the most significant predicted TF is known to be involved in the regulation process. Figure 2 shows that the tolerable amount of noise depends greatly on the quality of the TF signal in the set and on its size. For example, up to 50% of noise can be tolerated for the Rel/NF-*κ*B sample without altering the positive predictive value. Note also that for most data sets, noise levels higher than 50% result in the progressive loss of the true regulation signals.

**Figure 3:**
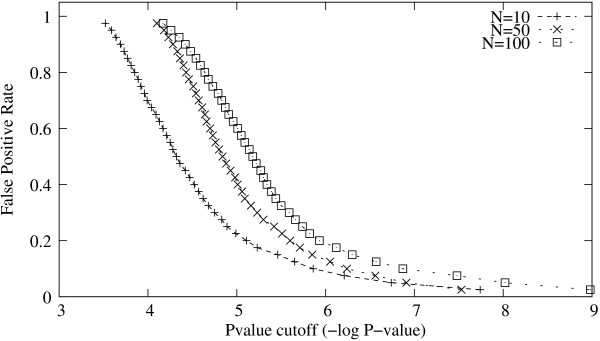
**Effect of P-value cutoff on the false positive error rate**. Various set sizes (5, 50, and 100 sequences) were used to evaluate the rate of false positive. The suggested P-value cutoffs for a fixed false positive rate of 10% are 10^-6 ^and 10^-8 ^for 5 and 100 sequences, respectively.

Lastly, we evaluated the specificity of TFM-Explorer under the extreme condition of there being only noise to identify (i.e., no signal present). This tested the level of the P-value that can be observed by chance. To achieve this we constructed a large number of sets of randomly selected genes that are not expected to share common functional TFBSs. Predictions returned by TFM-Explorer on these data sets are thus considered as false positive. To estimate the relationship between the false positive rate and the P-value cutoff, we generated 100 random sets of 10, 50, and 100 2-kb sequences. For each run, the ten most significant windows identified by TFM-Explorer and their associated P-value were retained. Figure 3 gives the percentage of trials that produced false positive predictions for each size of sample data set according to the chosen P-value cutoff. The first conclusion is that the cutoff must decrease with increasing sample size. For a fixed false positive rate of 10% (i.e., with no more than one false positive among the top ten), the optimal P-value cutoff was 10^-6 ^and 10^-7 ^for data sets containing 10 and 100 sequences, respectively. But for all data sets, a window with a P-value lower than 10^-10 ^can be considered significant.

## Conclusion

We have developed a new method for analyzing the regulatory regions of a set of genes sharing regulatory elements that can come from several species. Our method compares favorably with existing tools, such as TOUCAN, OTFBS, and oPOSSUM. We have also established that it can tolerate a high level of noise, which is a valuable property when dealing with experimental data derived from microarray experiments. One basic principle of the method is the use of the TSS as a reference element to handle gene upstream sequences. While this assumption proved to be valid for a variety of examples, it is insufficient for at least two reasons: (1) the correct position of the TSS is hard to annotate, and alternative splicing may lead to alternative TSSs; and (2) many regulatory elements show no spatial conservation relative to the TSS. Moreover, regulatory elements can even be found in introns or in 3'UTR. We believe that it would be useful to extend the method to other reference elements – such as highly conserved regions between species, or functional binding sites for a given regulatory protein – when searching for collaborative TFs.

## Availability and requirements

TFM-Explorer has been implemented in C/Python and is freely available upon request. It takes only a few seconds to execute on a standard workstation for a data sample of 20 genes with 2-kb-upstream promoter sequences while scanning for both TRANSFAC and JASPAR databases.

We also offer an easy-to-use Web-accessible server , which includes precomputed background models for human, mouse, and rat genomes. All annotated genes with RefSeq identifiers [29] available in the UCSC Genome Browser assembly [20] (release dates: hg18, March 2006; mm8, March 2006; rn3, June 2003) have been retrieved. This corresponds to 24 328, 19 343, and 8 314 genes for the human, mouse, and rat genomes, respectively. For all of these genes, promoter regions corresponding to 10 kb upstream and 5 kb downstream of the TSS were used to build background models. Potential TFBSs with a P-value of 0.001 have been exhaustively precomputed for all TRANSFAC and JASPAR vertebrate matrices. TFM-Explorer returns a ranked list of overrepresented TFBSs. Various types of complementary information are provided to enhance the understanding of the raw prediction, including the location of binding sites, the number of predicted binding sites per sequence in the window, the PWM composition and quality, and the correlation between hits and PWMs. The results can be exported as ASCII files for later use.

## Supplementary Material

Additional File 1 muscle gene set. list of RefSeq identifiers of human genes that have skeletal-muscle-specific expression. Click here for file

Additional File 2 NF-*κ*B gene set. list of RefSeq identifiers of human Rel/NF-*κ*B target genes. Click here for file

Additional File 3 H3 gene set. list of RefSeq identifiers of human, mouse, and rat genes that code for H3. Click here for file

## Figures and Tables

**Table 1:** Poisson chi-square goodness-of-fit test for the hit-count distribution. Percentage of PWMs for which the hit-count distribution (i.e., the number of putative TFBSs in a given sequence) is well modeled by a Poisson distribution according to the chi-square goodness-of-fit test, for three values of significance. Proximal upstream sequences from 1000 randomly selected human genes were used to compute the data listed.

**Matrices Database**	*α *> 0.1	*α *> 0.05	*α *> 0.01
**JASPAR**	72%	80%	87%
**TRANSFAC (public)**	68%	74%	83%

**Table 2:** Results for skeletal-muscle-specific human genes. Most significant TFBSs detected in the muscle data set by TFM-Explorer, TOUCAN, OTFBS, and oPOSSUM using sequences that were 2 kb upstream. The TRANSFAC vertebrate matrix collection was used with TOUCAN and OTFBS, and JASPAR vertebrate matrices were used with TFM-Explorer and oPOSSUM. TFs with experimentally verified sites in the data set are marked with *.

**TFM-Explorer**				
Rank		PWM	Window	P-value

1	*	SRF	[-0224: -0091]	7.869e-06
2	*	MEF2	[-0060: -0030]	2.350e-05
3	*	MZF_1-4	[-1431: -0576]	1.678e-04
4		Staf	[-1950: -1311]	2.539e-04
5		Irf-2	[-1892: -1592]	3.002e-04
6		NRF-2	[-0779: -0324]	3.180e-04
7		Brachyury	[-0307: -0048]	4.503e-04
8		Bsap	[-1911: -0978]	4.800e-04
9		cEBP	[-1733: -1679]	6.032e-04
10	*	MZF_5-13	[-1633: -1078]	7.141e-04

**TOUCAN**				

Rank		PWM		P-value

1		HEN1_01		8.567e-02
2	*	MEF2_02		1.021e-01
3		RSRFC4_01		1.129e-01
4		TAL1BETAITF2_01		1.311e-01
5		STAT5A_01		1.856e-01
6		TAL1BETAE47_01		2.322e-01
7		YY1_01		2.391e-01
8		STAT5B_01		2.534e-01
9	*	MEF2_03		3.056e-01
10		CDC5_01		3.134e-01

**OTFBS**				

Rank		PWM		P-value

1		YY1_02		2.047e-06
2	*	MZF1_02		2.763e-06
3	*	MEF2_02		9.493e-06

**oPOSSUM**				

Rank		PWM		P-value

1	*	MEF2		1.768e-04
2		Hen-1		3.730e-04
3		SRY		1.531e-03
4		c-MYB_l		1.780e-03
5		S8		2.983e-03
6		HFH-3		2.994e-03
7	*	SP1		3.220e-03
8	*	MZF_5-13		3.675e-03
9		Nkx		6.399e-03
10		RORalfa-2		7.747e-03

**Table 3:** Results for Rel/NF-*κ*B target genes. Most significant TFBSs detected in Rel/NF-*κ*B target genes set by TFM-Explorer, TOUCAN, OTFBS, and oPOSSUM. The data set comprised 99 human Rel/NF-*κ*B target genes that have experimentally verified binding sites. Both 2-kb-upstream sequences and 5-kb-downstream/5-kb-upstream sequences were used. The TRANSFAC vertebrate matrix collection was used with TFM-Explorer, TOUCAN, and OTFBS, and JASPAR vertebrate matrices were used with oPOSSUM. TFs of the Rel/NF-*κ*B family are marked with *. Other TFBSs (such as TATA-box) are also likely to be biologically valid.

**TFM-Explorer**, region -5000, 5000				
Rank		PWM	Window	P-value

1	*	NFKAPPAB65_01	[-0520: +0115]	8.875e-27
2	*	NFKAPPAB_01	[-0698: +0116]	1.026e-20
3	*	NFKB_C	[-0522: -0020]	9.148e-19
4		TATA_01	[-0056: -0010]	5.585e-18
5	*	NFKB_Q6	[-0537: +0092]	2.241e-16
6		TATA_C	[-0055: -0015]	4.128e-16
7	*	CREL_01	[-0501: -0020]	3.510e-15
8		CDXA_01	[-0071: -0018]	4.262e-15
9	*	NFKAPPAB50_01	[-0521: +0012]	8.601e-13
10		SP1_Q6	[-0094: -0043]	1.451e-11

**TFM-Explorer**, region -2000, 0				

Rank		PWM	Window	P-value

1	*	NFKAPPAB65_01	[-0520: -0019]	7.706e-27
2	*	NFKAPPAB_01	[-0698: -0019]	9.418e-20
3		TATA_0l	[-0056: -0023]	1.118e-19
4	*	NFKB_C	[-0522: -0020]	9.148e-19
5		TATA_C	[-0055: -0015]	4.128e-16
6	*	CREL_01	[-0501: -0020]	3.510e-15
7	*	CDXA_01	[-0071: -0018]	4.262e-15
8	*	NFKB_Q6	[-0537: -0021]	3.574e-14
9	*	NFKAPPAB50_01	[-0521: -0019]	1.066e-12
10		SP1_Q6	[-0094: -0043]	1.451e-11

**TOUCAN**, region -5000, 5000				

Rank		PWM		P-value

1		HFH3_01		0.0
2		BRACH_01		4.667e-01
3		RORA2_01		8.596e-01
4		NRSF_01		9.956e-01
5		E47_01		1.0
6		VMYB_01		1.0
7		AP4_01		1.0
8		MEF2_01		1.0
9		ELK1_01		1.0
10		EVI1_06		1.0

**TOUCAN**, region -2000, 0				

Rank		PWM		P-value

1	*	NFKAPPAB65_01		1.381e-05
2	*	NFKB_C		6.975e-05
3	*	NFKAPPAB_01		3.139e-04
4		ARP1_01		1.257e-03
5		SREBP1_01		6.795e-03
6	*	NFKB_Q6		4.683e-02
7	*	NFKAPPAB50_01		8.661e-02
8		RORA2_01		9.847e-02
9		E47_02		1.628e-01
10		HEN1_01		2.882e-01

**OTFBS**, region -5000, 5000				

Rank		PWM		P-value

				

**OTFBS**, region -2000, 0				

Rank		PWM		P-value

1		FOXJ2_01		6.097e-49
2		FOXD3_01		4.229e-45
3		HFH3_01		5.356e-41
4		HNF3B_01		7.352e-35
5		IK2_01		3.031e-20
6		SREBP1_01		1.969e-19
7	*	NFKAPPAB65_01		3.708e-19
8	*	NFKB_C		8.819e-19
9	*	CREL_01		2.571e-18
10		CHOP_01		1.004e-17

**oPOSSUM**, region -5000, 5000				

Rank		PWM		P-value

1	*	p65		1.941e-08
2	*	NF-kappaB		1.579e-05
3	*	c-REL		7.877e-05
4	*	p50		1.510e-04
5		c-FOS		6.236e-04
6		Irf-1		3.301e-03
7		MZF_5-13		5.543e-03
8		MZF_1-4		7.967e-03
9		NRF-2		2.933e-02
10		SPI-B		3.239e-02

**oPOSSUM**, region -2000, 0				

Rank		PWM		P-value

1	*	p65		1.333e-14
2	*	NF-kappaB		3.234e-11
3	*	c-REL		4.835e-09
4	*	p50		3.272e-07
5		SPI-B		5.137e-05
6		c-FOS		1.519e-04
7		Elk-1		2.329e-04
8		deltaEF1		2.877e-04
9		MZF_1-4		3.731e-04
10		Irf-1		6.815e-04

**Table 4:** Results for the H3 gene set. Most significant TFBSs detected in the H3 data set by TFM-Explorer, TOUCAN, OTFBS, and oPOSSUM using sequences that were 2 kb upstream. The TRANSFAC vertebrate matrices were used with TOUCAN, OTFBS, and TFM-Explorer. oPOSSUM was unable to produce results from this data set. TFs with experimentally verified sites in the set are marked with *.

**TFM-Explorer**				
Rank		PWM	Window	P -value

1	*	NFY_C	[-1375: -0039]	4.757e-24
2	*	OCT1_04	[-0588: -0022]	1.537e-20
3	*	NFY_Q6	[-1318: -0039]	4.026e-16
4	*	OCT1_07	[-0574: -0025]	7.932e-14
5		XFD1_01	[-0890: -0025]	2.253e-13
6		PBX1_02	[-0491: -0040]	2.737e-13
7		SRY_02	[-0895: -0015]	1.803e-12
8		MEF2_04	[-0482: -0038]	1.826e-12
9		HNF1_01	[-0642: -0097]	7.089e-12
10		EVI1_04	[-0417: -0040]	9.277e-12

**TOUCAN**				

Rank		PWM		P-value

1	*	NFY_01		1.364e-08
2	*	OCT1_01		1.854e-05
3		GFI1_01		4.506e-05
4		TATA_01		1.315e-03
5	*	CAAT_01		1.781e-03
6	*	OCT_C		1_018e-02
7		MEF2_02		1.041e-02
8		MEF2_03		1.041e-02
9		NFY_C		1.633e-02
10		CART1_01		2.569e-02

**OTFBS**				

Rank		PWM		P-value

1		IRF1_01		5.099e-26
2		HFH3_01		6.865e-22
3		FOXJ2_01		1.606e-21
4		MEF2_01		6.896e-20
5		HNF3B_01		1.165e-18
6		MEF2_04		1.243e-18
7		FOXD3_01		3.698e-18
8		MEF2_02		2.964e-15
9		XFD1_01		8.016e-15
10	*	NFY_C		6.396e-14
